# The important choice of reference environment in microevolutionary climate response predictions

**DOI:** 10.1002/ece3.8836

**Published:** 2022-04-17

**Authors:** Rolf Ergon

**Affiliations:** ^1^ University of South‐Eastern Norway Porsgrunn Norway

**Keywords:** climate response predictions, environmental zero point, microevolution, plasticity, prediction error minimization, reference environment

## Abstract

It is well documented that individuals of wild populations can adjust to climate change by means of phenotypic plasticity, but few reports on adaptation by means of genetically based microevolution caused by selection. Disentanglement of these separate effects requires that the reference environment (the environmental zero point) is defined, and this should not be done arbitrarily. The problem is that an error in the reference environment may lead to large errors in predicted microevolution. Together with parameter values and initial mean trait values, the reference environment can be estimated from environmental, phenotypic and fitness data. A prediction error method for this purpose is described, with the feasibility shown by simulations. As shown in a toy example, an estimated reference environment may have large errors, especially for small populations. This may still be a better choice than use of an initial environmental value in a recorded time series, or the mean value, which is often used. Another alternative may be to use the mean value of a past and stationary stochastic environment, which the population is judged to have been fully adapted to, in the sense that the expected geometric mean fitness was at a global maximum. Exceptions are cases with constant phenotypic plasticity, where the microevolutionary changes per generation follow directly from phenotypic and environmental data, independent of the chosen reference environment.

## Graphical abstract

Microevolutionary climate response predictions must be based on the best possible knowledge of the environmental reference (zero point).

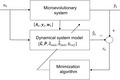

## 1 INTRODUCTION

Wild populations respond to changing environments by means of phenotypic plasticity and microevolution, and especially climate change responses have been extensively studied. The aim is then to disentangle phenotypic changes owing to genetically based microevolution caused by natural selection, and changes due to individual phenotypic plasticity. Relying on 11 review articles, including reviews of altogether 66 field studies, Merilä and Hendry (2014) arrived at the conclusion that evidence for genetic adaptation to climate change has been found in some systems, but that such evidence is relatively scarce. They also concluded that more studies were needed, and that these must employ better inferential methods. The aim of the present article is to give a contribution in that last respect.

It is obvious that for all evolutionary systems with interval‐scaled environmental variables $u_{t}$, as, for example, temperature in °C, a suitable zero point (reference environment) $u_{\text{ref}}$ must be chosen, and as argued in Section 2, this should not be done arbitrarily. A zero point is in general defined as “the point on a scale that denotes zero and from which positive and negative readings can be made” (Collins English Dictionary). The problem is that an error in the reference environment may lead to large errors in predicted microevolution. In most cases where the environmental variable is, for example, a temperature, the reference environment should not, for example, be set to 0°C (or 0°F). Neither should it, without further consideration, be set to the initial or mean environmental value of a specific time series. It appears that the need for a proper reference environment definition, and thus also an environmental cue definition, has been largely ignored in the reviewed studies referred to in Merilä and Hendry (2014).

The present article is an attempt to clarify some important questions relating to reference environments, and for that purpose a method for model‐based predictions of microevolutionary changes is also proposed. This method is based on parameter estimation by means of prediction error minimization, including estimation of reference environment and initial mean values of quantitative traits.

For a discussion on the general microevolution versus plasticity disentanglement problem, we may for simplicity assume the intercept‐slope individual reaction norm model:

(1) $$y_{i,t}=a_{i,t}+v_{i,t}+\left(b_{i,t}+\eta_{i,t}\right)\left(u_{t}‐u_{\text{ref}}\right),$$

where $u_{t}‐u_{\text{ref}}$ and $y_{i,t}$ are the environmental cue and the individual phenotypic value, respectively, as functions of time t measured in generations. Here, $a_{i,t}$ and $b_{i,t}$ are the additive genetic components of the intercept and plasticity slope, respectively, while $v_{i,t}$ and $\eta_{i,t}$ are independent iid zero mean normal non‐additive effects. As done in Lande (2009) and Ergon and Ergon (2017), we may consider the individual reaction norm intercept $a_{i,t}+v_{i,t}$, and the individual plasticity slope $b_{i,t}+\eta_{i,t}$, as two quantitative traits in their own right. Microevolution thus involves changes in the mean trait values ${\bar{a}}_{t}$ and ${\bar{b}}_{t}$ from generation to generation. The generations are here assumed to be non‐overlapping.

From Equation (1) follows the mean trait reaction norm model, ${\bar{y}}_{t}={\bar{a}}_{t}+{\bar{b}}_{t}\left(u_{t}‐u_{\text{ref}}\right)$, and from this simple equation follows the basic questions discussed in this article. How can $u_{\text{ref}}$ be estimated, and how can the evolution of ${\bar{a}}_{t}$ and ${\bar{b}}_{t}$ be predicted, provided that $u_{t}$ and $y_{i,t}$ are known? And how will the predictions be affected by errors in the estimated or assumed value ${\hat{u}}_{\text{ref}}$? It turns out that in order to answer these questions we also need information on individual fitness values $W_{i,t}$.

The reference environment $u_{\text{ref}}$ is determined by the environment at which the phenotypic variance has its minimum, as defined in more detail in Section 2, and as discussed in Ergon and Ergon (2017) and Ergon (2018). In theoretical work, it is often assumed that the population has fully adapted to a stationary stochastic environment with a given mean value, such that the expected geometric mean fitness is at a global maximum, and the reference environment is then set to zero (Chevin & Lande, 2015; Lande, 2009). Although there is nothing wrong with this theoretical approach, it disguises the underlying problem discussed here, and $u_{\text{ref}}$ is therefore included in Equation (1). This formulation also makes it possible to distinguish between the environment as such and the environmental cue. In some cases, it may be possible to determine the reference environment experimentally, see, for example, Fossen et al. (2018), but that may obviously be difficult for wild populations.

When the environmental cue $u_{t}‐u_{\text{ref}}$ changes over time, the mean trait values ${\bar{a}}_{t}$ and ${\bar{b}}_{t}$ as follow from Equation (1) may evolve due to selection, and as a result also the mean phenotypic value ${\bar{y}}_{t}$ will evolve (Lande, 2009). Without changes due to selection, that is, if the mean trait values ${\bar{a}}_{t}$ and ${\bar{b}}_{t}$ are constant, the value of ${\bar{y}}_{t}$ may still change when $u_{t}‐u_{\text{ref}}$ changes, as also follows from Equation (1).

Section 2 discusses several aspects of the general microevolution versus plasticity disentanglement problem. First, a definition of the reference environment is given. Second, it is shown how the mean trait values ${\bar{a}}_{t}$ and ${\bar{b}}_{t}$, and thus also ${\bar{y}}_{t}$, evolve as functions of the environmental cue $u_{t}‐u_{\text{ref}}$ and the phenotypic selection gradient $\beta_{y,t}$. Third, it is shown how $u_{\text{ref}}$ and $\beta_{y,t}$, as well as initial mean trait values and the parameter values in the G matrix, can be estimated by means of a prediction error minimization method (Ljung, 2002), using data from known time series of $u_{t}$ and $y_{i,t}$, as well as of individual fitness values $W_{i,t}$. Fourth, it is discussed why it may be difficult to estimate $u_{\text{ref}}$, as revealed by simulations, and which consequences errors in estimated values of $u_{\text{ref}}$ will have. Exceptions are cases with constant phenotypic plasticity, where the microevolutionary changes per generation follow directly from phenotypic and environmental data, independent of the chosen reference environment.

It must be underlined that the theory in Section 2 assumes that the phenotypic trait $y_{i,t}$ in Equation (1) is not correlated with other phenotypic traits having causal effects on fitness, see Morrissey et al. (2010) for a discussion. Also note that the need for a proper reference environment is not specific for the simple case according to Equation (1).

Simulations in Section 3 make use of a toy example, utilizing the intercept‐slope reaction norm model in Equation (1). The environmental input $u_{t}$ is here a noisy positive trend in spring temperature, while the individual phenotypic values $y_{i,t}$ are the clutch initiation dates for a certain bird species. The toy example also assumes that the individual (mid‐parent) fitness values $W_{i,t}$ are the numbers of offspring. The essential questions are how microevolutionary changes in mean intercept and plasticity slope can be predicted, and how these predictions are affected by errors in the reference environment $u_{\text{ref}}$ in Equation (1). The simulations show that errors in the estimated or assumed value of $u_{\text{ref}}$ may cause large mean trait prediction errors. They also show the feasibility of the proposed parameter estimation method.

Finally, follows a discussion in Section 4. Derivations of prediction equations, simulation results with modeling error and increased population size, and a short comparison with BLUP/REML parameter estimation are given in Appendices S1–S4.

## 2 THEORY AND METHODS

### 2.1 Example system

For a study of the general reference environment problem, and for a test of the proposed parameter estimation method, we may consider a true evolutionary system based on Equation (1),

(2a) $${\bar{y}}_{t}={\bar{a}}_{t}+{\bar{b}}_{t}\left(u_{t}‐u_{\text{ref}}\right),$$

(2b) $$\left[\begin{array}{c} \Delta {\bar{a}}_{t}\\ \Delta {\bar{b}}_{t} \end{array}\right]=\frac{1}{{\bar{W}}_{t}}{\mathrm{GP}}^{‐1}\left[\begin{array}{c} \text{cov}\left(W_{i,t},a_{i,t}+v_{i,t}\right)\\ \text{cov}\left(W_{i,t},b_{i,t}+\eta_{i,t}\right) \end{array}\right]$$

with the additive genetic covariance matrix $G=\left[\begin{array}{cc} G_{\mathrm{aa}} & G_{\mathrm{ab}}\\ G_{\mathrm{ab}} & G_{\mathrm{bb}} \end{array}\right]$ , and the phenotypic covariance matrix $P=\left[\begin{array}{cc} G_{\mathrm{aa}}+\sigma_{v}^{2} & G_{\mathrm{ab}}\\ G_{\mathrm{ab}} & G_{\mathrm{bb}}+\sigma_{\eta }^{2} \end{array}\right]$ . Here, Equation (2b) is the multivariate breeder's equation (Lande, 1979), where $W_{i,t}$ is found from any given fitness function. It is assumed that the phenotypic trait $y_{i,t}$ in Equation (1) is not correlated with other phenotypic traits having causal effects on fitness, and that generations are non‐overlapping.

### 2.2 Reference environment and environmental cue definitions

As discussed in the Introduction, there is a need for reference environment and environmental cue definitions:

Definition 1 Assuming a single environmental variable $u_{t}$, and given a reaction norm model, the reference environment is as follows:

(3) $$u_{\text{ref}}=u_{0}+f\left(\text{reaction norm parameter values}\right),$$

where $u_{0}$ is the environment at which the phenotypic variance is at a minimum, and where the covariance between the plastic phenotypic value and reaction norm slope is equal to 0. Here, $f\left(\text{reaction norm parameter values}\right)$ is a correction term that may be 0.

Definition 2 With $u_{\text{ref}}$ according to Definition 1, the environmental cue is given by $u‐u_{\text{ref}}$.

For the reaction norm model (1) we find, for example, (using $u^{\prime }=u‐u_{\text{ref}}$)

(4a) $$\text{cov}\left(y,b\right)=E\left[\left(a‐\bar{a}+\nu +\left(b‐\bar{b}\right)u^{\prime }+\eta u^{\prime }\right)\left(b‐\bar{b}+\eta \right)\right]=G_{\mathrm{ab}}+\left(G_{\mathrm{bb}}+\sigma_{\eta }^{2}\right)u^{\prime },$$

which by setting $\text{cov}\left(y,b\right)=0$ and $u^{\prime }=u_{0}‐u_{\text{ref}}$ gives the reference environment

(4b) $$u_{\text{ref}}=u_{0}+\frac{G_{\mathrm{ab}}}{G_{\mathrm{bb}}+\sigma_{\eta }^{2}}.$$

**FIGURE 1:**
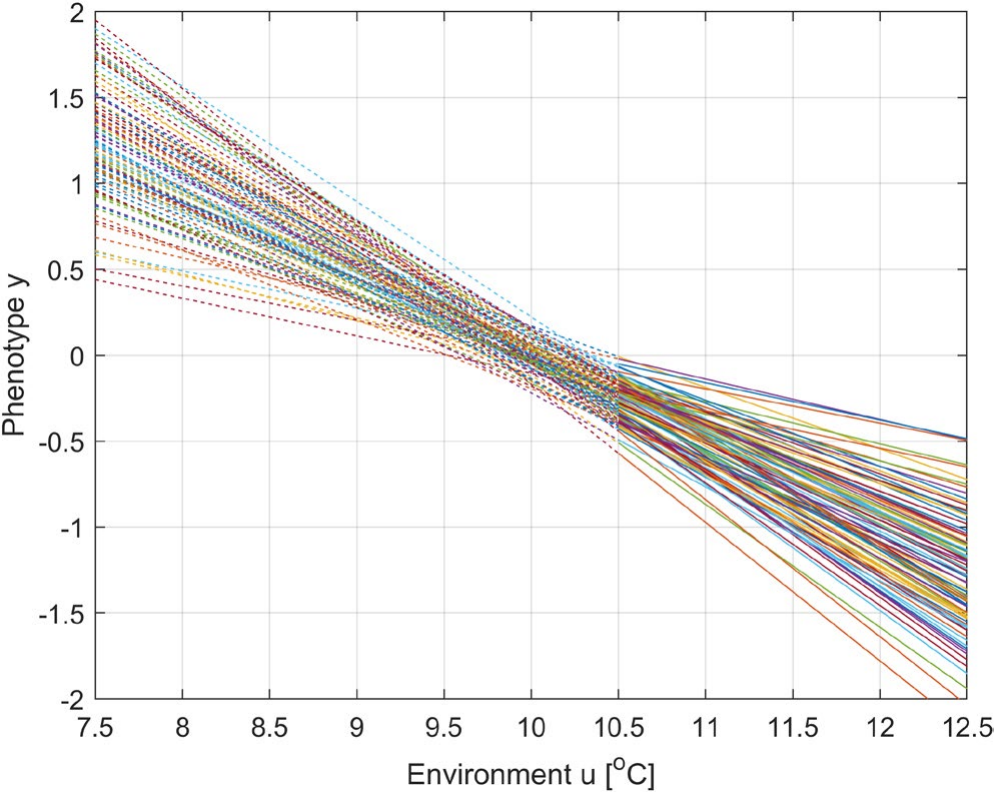
Reaction norms for 100 individuals in a population according to Equation (1), with $G_{\mathrm{aa}}+\sigma_{v}^{2}=0.05$, $G_{\mathrm{bb}}+\sigma_{\eta }^{2}=0.02,$ and $G_{\mathrm{ab}}=0$. The reference environment is $u_{\text{ref}}=10$°C, which since $G_{\mathrm{ab}}=0$ also is the temperature $u_{0}$ to which the population is fully adapted. The mean trait values are ${\bar{a}}_{t}=0$ and ${\bar{b}}_{t}=‐0.5$. Solid lines indicate the range of data used for parameter estimation and mean trait predictions in simulations. Note that $u_{\text{ref}}=u_{0}=10$°C is not within that range

For $G_{\mathrm{ab}}=0,$ the reference environment is thus the environment where the phenotypic variance is minimized (see Figure 1 for illustration). This is also the environment where the expected geometric mean fitness has a global maximum, and thus the environment the population is fully adapted to. In this environment the environmental cue will be 0.

### 2.3 Mean trait prediction equations

A fundamental equation for mean trait predictions follows from Equation (2a) as

(5) $$\Delta {\bar{y}}_{t}=\Delta {\bar{a}}_{t}+\Delta {\bar{b}}_{t}\left(u_{t+1}‐u_{\text{ref}}\right)+{\bar{b}}_{t}\Delta u_{t},$$

where $\Delta u_{t}=u_{t+1}‐u_{t}$, $\Delta {\bar{a}}_{t}={\bar{a}}_{t+1}‐{\bar{a}}_{t}$, $\Delta {\bar{b}}_{t}={\bar{b}}_{t+1}‐{\bar{b}}_{t},$ and $\Delta {\bar{y}}_{t}={\bar{y}}_{t+1}‐{\bar{y}}_{t}$ are changes per generation. From this follows that the value of $u_{\text{ref}}$ has nothing to say in special cases with constant phenotypic plasticity slopes, that is, when $\Delta {\bar{b}}_{t}=0$. In such cases, we simply have $\Delta {\bar{y}}_{t}=\Delta {\bar{a}}_{t}+\bar{b}\Delta u_{t}$, where $\bar{b}$ is constant, or only $\Delta {\bar{y}}_{t}=\bar{b}\Delta u_{t},$ if ${\bar{a}}_{t}$ does not evolve.

As shown in Appendix S1, Equation (5) leads to equations for $\Delta {\bar{a}}_{t}$ and $\Delta {\bar{b}}_{t}$ as functions of the phenotypic selection gradient $\beta_{y,t}$,

(6a) $$\Delta {\bar{a}}_{t}=\left(G_{\mathrm{aa}}+G_{\mathrm{ab}}\left(u_{t}‐u_{\text{ref}}\right)\right)\beta_{y,t}$$

and

(6b) $$\Delta {\bar{b}}_{t}=\left(G_{\mathrm{ab}}+G_{\mathrm{bb}}\left(u_{t}‐u_{\text{ref}}\right)\right)\beta_{y,t},$$

where

(6c) $$\beta_{y,t}=\frac{1}{{\bar{W}}_{t}}{\left(P_{\mathrm{aa}}+2G_{\mathrm{ab}}\left(u_{t}‐u_{\mathrm{ref}}\right)+P_{\mathrm{bb}}{\left(u_{t}‐u_{\text{ref}}\right)}^{2}\right)}^{‐1}\text{cov}\left(W_{i,t},y_{i,t}\right).$$

In addition to time series of $u_{t}$ and $y_{i,t}$, we thus need parameter values for $u_{\text{ref}}$, $G_{\mathrm{aa}}$, $G_{\mathrm{ab}}$, $G_{\mathrm{bb}}$, $\sigma_{v}^{2}$, and $\sigma_{\eta }^{2}$, and a time series of individual fitness values $W_{i,t}$. For mean trait predictions, we also need initial values. Note that these equations are valid only when the genetic relationship matrix is a unity matrix (Ch. 26, Lynch & Walsh, 1998).

### 2.4 Prediction error minimization method

**FIGURE 2:**
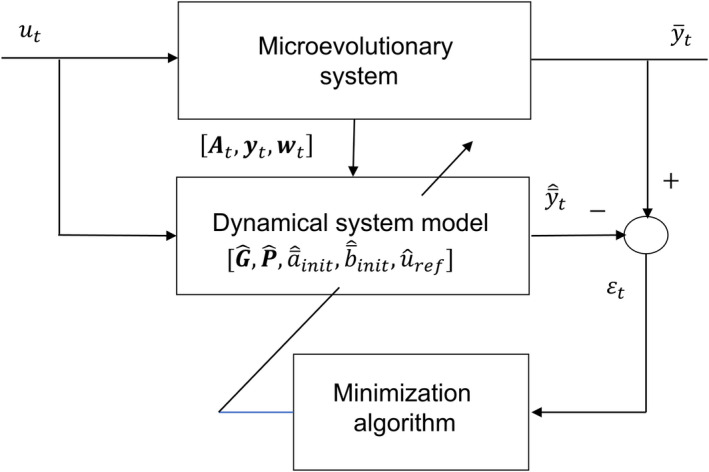
Block diagram of microevolutionary PEM, with dynamical tuning model based on an intercept‐slope reaction norm model with mean traits ${\bar{a}}_{t}$ and ${\bar{b}}_{t}$. Here, $u_{t}$ and ${\bar{y}}_{t}$ are the known environmental input and the known mean phenotypic value at time t, respectively. $A_{t}$ is the additive genetic relationship matrix, which here is assumed to be $A_{t}=I$, while $y_{t}$ and $w_{t}$ are vectors of individual phenotypic and relative fitness values, respectively. The $\hat{G}$ and $\hat{P}$ matrices include the system parameters, while ${\hat{\bar{a}}}_{\text{init}}$, ${\hat{\bar{b}}}_{\text{init}}$, and ${\hat{u}}_{\text{ref}}$ are the initial mean trait values and the reference environment, respectively. Assuming data over T generations, all these model parameters are tuned until $\sum_{t=1}^{T}\varepsilon_{t}^{2}=\sum_{t=1}^{T}{\left({\bar{y}}_{t}‐{\hat{\bar{y}}}_{t}\right)}^{2}$ is minimized, with ${\bar{y}}_{t}={\hat{\bar{y}}}_{1}=0$ and ${\hat{\bar{a}}}_{1}=‐{\hat{\bar{b}}}_{1}\left(u_{1}‐{\hat{u}}_{\text{ref}}\right)$

From predicted mean intercept and plasticity slope values found by Equations (6a, 6b) follow predicted values of ${\bar{y}}_{t}$ from Equation (2a). The prediction equations can thus be used for parameter estimation in a prediction error minimization method (PEM), as shown in Figure 2. As follows from Equations ((6a), (6b), (6c)), we can then set $G_{\mathrm{aa}}$ to any value, and estimate $G_{\mathrm{ab}}$, $G_{\mathrm{bb}}$, $\sigma_{v}^{2}$, and $\sigma_{\eta }^{2}$ relative to that value.

### 2.5 Effects of errors in the reference environment

With a reference environment ${\hat{u}}_{\text{ref}}$ instead of $u_{\text{ref}}$, predictions based on Equation (2a) can be written

(7) $${\hat{\bar{y}}}_{t}={\hat{\bar{a}}}_{t}+{\hat{\bar{b}}}_{t}\left(u_{t}‐{\hat{u}}_{\text{ref}}\right)={\hat{\bar{a}}}_{t}‐{\hat{\bar{b}}}_{t}\left({\hat{u}}_{\text{ref}}‐u_{\text{ref}}\right)+{\hat{\bar{b}}}_{t}\left(u_{t}‐u_{\text{ref}}\right),$$

where ${\hat{\bar{a}}}_{t}$ and ${\hat{\bar{b}}}_{t}$ are found from Equations (6a, 6b) with use of estimated parameter values, assuming initial values known.

For small values of $G_{\mathrm{bb}}$, that is, when $G_{\mathrm{bb}}\to 0$ and $G_{\mathrm{ab}}\to 0$, it follows from Equations ((6a), (6b), (6c)), that $\Delta {\bar{a}}_{t}$ is independent of $u_{\text{ref}}$, and that ${\bar{b}}_{t}$ is constant. This results in $\Delta {\hat{\bar{a}}}_{t}=G_{\mathrm{aa}}/\left(G_{\mathrm{aa}}+{\hat{\sigma }}_{v}^{2}\right)$, such that only ${\hat{\sigma }}_{v}^{2}$ must be tuned in order to minimize $\sum_{t=1}^{T}{\left({\bar{y}}_{t}‐{\hat{\bar{y}}}_{t}\right)}^{2}$. In this case, an error in ${\hat{u}}_{\text{ref}}$ has very little effect on the change in ${\hat{\bar{a}}}_{t}$ per generation, as also follows from Equation (5).

For larger values of $G_{\mathrm{bb}}$, the predicted change per generation $\Delta {\hat{\bar{a}}}_{t}$ will be affected by an error in ${\hat{u}}_{\mathrm{ref}}$, and with $G_{\mathrm{ab}}=0$ good predictions ${\hat{\bar{y}}}_{t}\approx {\bar{y}}_{t}$ for t=1 to T can then only be obtained by parameter tuning such that ${\hat{\bar{b}}}_{t}\approx {\bar{b}}_{t}$ over all generations. That is possible because $u_{\text{ref}}$ appears in both nominator and denominator of Equation (6b). According to Equation (7) we then find ${\hat{\bar{a}}}_{t}\approx {\bar{a}}_{t}+{\hat{\bar{b}}}_{t}\left({\hat{u}}_{\text{ref}}‐u_{\text{ref}}\right)$, which as shown in Section 3 may result in large errors in predicted changes in ${\bar{a}}_{t}$ over time.

Equation (7) also shows why it may be difficult to estimate $u_{\text{ref}}$ from data based on small populations. The reason is that minimization of $\sum_{t=1}^{T}{\left({\bar{y}}_{t}‐{\hat{\bar{y}}}_{t}\right)}^{2}$ results in ${\hat{\bar{a}}}_{t}‐{\hat{\bar{b}}}_{t}\left({\hat{u}}_{\mathrm{ref}}‐u_{\mathrm{ref}}\right)\approx {\bar{a}}_{t}$ and ${\hat{\bar{b}}}_{t}\approx {\bar{b}}_{t}$ over all generations, also if there is a large error in ${\hat{u}}_{\text{ref}}$.

### 2.6 Effects of modeling errors

Modeling errors will obviously affect predictions of the mean traits. As an example, simulations with the true individual model

(8) $$y_{i,t}=a_{i,t}+v_{i,t}+\left(b_{i,t}+\eta_{i,t}\right)\left(u_{t}‐c_{i,t}‐\gamma_{i,t}\right),$$

are included in Appendix S2. Here, $c_{i,t}+\gamma_{i,t}$ is a perception trait, as discussed in Ergon and Ergon (2017).

## 3 SIMULATION RESULTS

### 3.1 Description of toy example

In the toy example, the environmental input ($u_{t}$) is a noisy positive trend in spring temperature, resulting in a noisy negative trend in mean clutch initiation date (${\bar{y}}_{t}$) for a certain bird species, approximately as in figure 2 in Bowers et al. (2016). The individual phenotypic values are discrete, with days as unit. The individual (mid‐parent) fitness values ($W_{i,t}$) are integers from 0 to 10, with number of fledglings as unit. Generations are assumed to be non‐overlapping, and the population size is assumed to be constant. Data for $u_{t}$, $y_{i,t},$ and $W_{i,t}$ are generated over 60 generations, where the positive temperature trend begins at generation 10. The population is assumed to be fully adapted to the mean spring temperature 10°C before generation 10, which is thus the reference environment $u_{\text{ref}}$, but only data from generations 31 to 60 are used for parameter estimation and mean trait predictions. Note that 10°C may not be within the range of input data used for parameter estimation (depending on realization). The essential questions are how $u_{\text{ref}}$ may be estimated, how well microevolutionary changes in mean intercept and plasticity slope over generations 31 to 60 can be predicted by means of the PEM method in Figure 2, and how errors in the estimated or assumed value of $u_{\text{ref}}$ will affect the predictions.

### 3.2 True model, fitness function, and environmental input signals

Assume that what we consider to be true mean responses, ${\bar{y}}_{t}$, ${\bar{a}}_{t},$ and ${\bar{b}}_{t}$, are generated by the state‐space model (2a, 2b). Here, $G_{\mathrm{ab}}=0$ in the true system but left as a free parameter in the tuning model in Figure 2. The individual effects $a_{i,t}$, $b_{i,t}$, $v_{i,t}$ and $\eta_{i,t}$ are at each generation drawn from populations with normal distributions around ${\bar{a}}_{t}$,${\bar{b}}_{t},$ 0 and 0, respectively.

The individual fitness function is assumed to be rounded values of

(9) $$W_{i,t}=10\cdot \exp\left(‐{\left(y_{i,t}‐\theta_{t}\right)}^{2}/2\omega^{2}\right),$$

where $\theta_{t}$ is the phenotypic value that maximizes fitness, while $\omega^{2}=10$. The discrete values of $W_{i,t}$ (number of fledglings) are thus integers from 0 to 10.

Also assume a stationary or slowly varying mean $\mu_{U,t}$ of a stochastic environment, with added iid zero mean normal random variations $u_{n,t}$ with variance $\sigma_{U_{n}}^{2}$, that is, $u_{t}=\mu_{U,t}+u_{n,t}$, and that the population is fully adapted to a stationary stochastic environment with $\mu_{U,t}=u_{\text{ref}}=u_{0}=10^{\circ }C$ (as in Figure 1). In a corresponding way, assume that $\theta_{t}=\mu_{\Theta ,t}+\theta_{n,t}$, where $\theta_{n,t}$ is iid zero mean normal with variance $\sigma_{\Theta_{n}}^{2}$, and where $u_{n,t}$ and $\theta_{n,t}$ are correlated with covariance $\sigma_{\Theta_{n}U_{n}}$. Following Lande (2009), we may assume that juvenile birds of generation t are exposed to the environment $u_{t‐\tau }$ during a critical period of development a fraction of a generation before the adult phenotype is expressed and subjected to natural selection. We will define $\theta_{t}=‐2\left(u_{t}‐10\right)$, which implies a linear relationship $\mu_{\Theta ,t}=‐2\left(\mu_{U,t}‐10\right)$, variances $\sigma_{\Theta_{n}}^{2}=4\sigma_{U_{n}}^{2}$, and covariance $\sigma_{\Theta_{n}U_{n}}=‐2\rho_{\tau }\sigma_{U_{n}}^{2}$, where $\rho_{\tau }$ is the autocorrelation of background environmental fluctuations. We will assume $\sigma_{U_{n}}^{2}=0.5$ and $\rho_{\tau }=0.25$. The optimal value of the mean plasticity slope in a stationary stochastic environment is then ${\bar{b}}_{\mathrm{opt}}=\sigma_{\Theta_{n}U_{n}}/\sigma_{U_{n}}^{2}=‐2\rho_{\tau }=‐0.5$ (as in Figure 1) (Ergon & Ergon, 2017).

**FIGURE 3:**
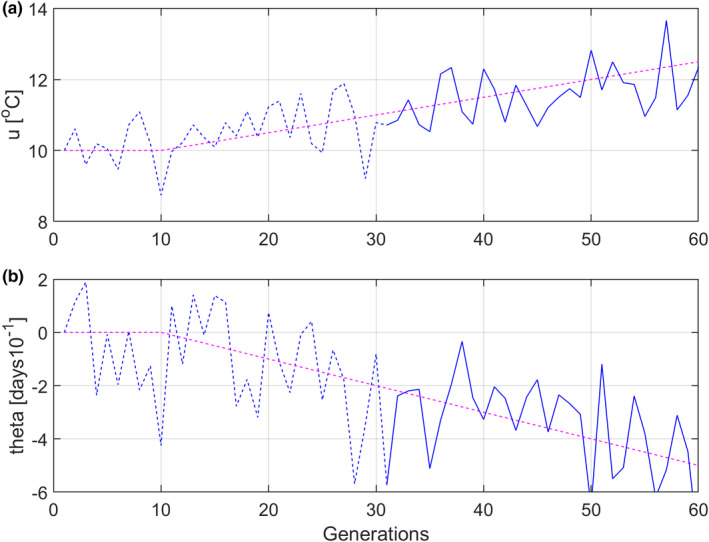
Noisy ramp functions $u_{t}$ (panel a) and $\theta_{t}$ (panel b), with $u_{\text{ref}}=10$, $\mu_{\Theta ,t}=‐2\left(\mu_{U,t}‐10\right)$, $\sigma_{U_{n}}^{2}=0.5$, $\sigma_{\Theta_{n}}^{2}=2$, and $\sigma_{U_{n},\Theta_{n}}=‐0.25.$ The solid parts of the curves indicate data used for parameter estimation and mean trait predictions (compare with Figure 1, where reaction norms for $u_{t}>10.5$ are indicated by solid lines)

Further assume that $u_{t}$ and $\theta_{t}$ are noisy ramp functions as shown in Figure 3, with the ramp in $\mu_{U,t}$ starting from 10°C at t=10 generations. The choice of a negative trend in $\theta_{t}$, and thus in ${\bar{y}}_{t}$, results in earlier clutch initiation dates as a result of the positive temperature trend.

**FIGURE 4:**
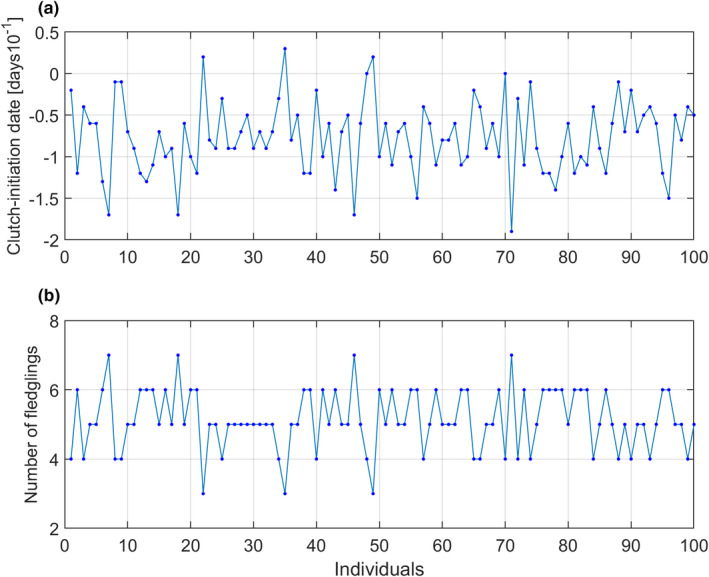
Individual clutch initiation dates $y_{i,t}$, with a range from ‐19 to 3 days (phenotypic values, Panel a), and number of fledglings $W_{i,t}$ (fitness, Panel b), at a generation where the population is under directional selection toward earlier clutch initiation dates. As indicated by dots, the number of offspring are integers, while the phenotypic values are integers divided by 10 (i.e., days)

Figure 4 shows typical individual phenotypic (clutch initiation date) and fitness (number of fledglings) values for the true model with population size N=100 at generation 45 in Figure 3. The figure shows that the most negative (earliest) dates give the highest number of offspring, and the population is thus under directional selection toward earlier clutch initiation dates. The zero‐point date is the mean clutch initiation date before the positive temperature trend sets in at generation 10 in Figure 3, when the population is assumed to be under stabilizing selection and fully adapted to the stationary stochastic temperature.

### 3.3 Parameter estimation and mean trait prediction results

Parameter estimation and mean trait prediction results were found by use of the MATLAB function *fmincon* in the PEM method in Figure 2. Results with use of input–output data from t=31 to 60 with population size N=100 are given in Table 1. The relative errors in total change in predictions over 30 generations are included, computed as $\Delta_{30}^{\text{error}}{\hat{\bar{a}}}_{t}\%=100\left(\Delta_{30}{\hat{\bar{a}}}_{t}‐\Delta_{30}{\bar{a}}_{t}\right)/\Delta_{30}{\bar{a}}_{t}$ etc., where $\Delta_{30}{\hat{\bar{a}}}_{t}={\hat{\bar{a}}}_{60}‐{\hat{\bar{a}}}_{31}$ and $\Delta_{30}{\bar{a}}_{t}={\bar{a}}_{60}‐{\bar{a}}_{31}$. The final values $\sum \varepsilon_{t,\text{final}}^{2}$ of $\sum_{t=31}^{60}{\left({\bar{y}}_{t}‐{\hat{\bar{y}}}_{t}\right)}^{2}$ are also included, as they indicate the degree of optimization success. Results are presented as mean values and standard errors, Mean ± SE, based on 100 repeated simulations with different realizations of random inputs.

**TABLE 1:** Estimation and prediction results with true system responses generated by means of Equations (2a, 2b) and (9). Results are for cases with population size N=100 and perfect observations of $y_{i,t}$ and $W_{i,t}$, and they are based on 100 simulations with different realizations of all random input variables

Parameter etc.	True value	Results Case 1	Results Case 2	Results Case 3
${\hat{G}}_{\mathrm{bb}}$	0.01	0.0103 ± 0.0023	0.0099 ± 0.0054	0.0065 ± 0.0023
${\hat{G}}_{\mathrm{ab}}$	0	0.0033 ± 0.0141	0.0028 ± 0.0152	0.0067 ± 0.0022
${\hat{\sigma }}_{v}^{2}$	0.025	0.0341 ± 0.0141	0.0301 ± 0.0348	0.0264 ± 0.0040
${\hat{\sigma }}_{\eta }^{2}$	0.01	0.0118 ± 0.0091	0.0123 ± 0.0115	0.0157 ± 0.0037
${\hat{\bar{b}}}_{31}$	–	−0.4965 ± 0.0086	−0.4969 ± 0.0083	−0.4985 ± 0.0080
${\hat{u}}_{\mathrm{ref}}$	10	10	9.9210 ± 0.6553	11
$\sum \varepsilon_{t,\mathrm{final}}^{2}$	–	10^−5^ (23 ± 10)	10^−5^ (23 ± 10)	10^−5^ (29 ± 11)
$\Delta_{30}^{\mathrm{error}}{\hat{\bar{a}}}_{t}\%$	–	1 ± 4	−5 ± 42	68 ± 8
$\Delta_{30}^{\mathrm{error}}{\hat{\bar{b}}}_{t}\%$	–	−1 ± 3	0 ± 3	−4 ± 4
$\Delta_{30,\mathrm{corr}}^{\mathrm{error}}{\hat{\bar{a}}}_{t}\%$	–	1 ± 4	0 ± 5	−4 ± 5

Note

Case 1: ${\hat{u}}_{\text{ref}}=10$ (the true value). Case 2: ${\hat{u}}_{\text{ref}}$ as a free variable. Case 3: ${\hat{u}}_{\text{ref}}=11$ (expected initial value in optimization data). Here, 9% of the simulations were discarded because $\sum \varepsilon_{t,\text{final}}^{2}>0.001$.

Given the model in Equations (2a, 2b) and (9), there are in all six parameter values to be estimated (while ${\hat{\bar{a}}}_{31}$ follows from Equation (2a) with ${\hat{\bar{y}}}_{31}$ set to 0). In the optimizations, the initial values of ${\hat{G}}_{\mathrm{bb}}$, ${\hat{G}}_{\mathrm{ab}}$, ${\hat{\sigma }}_{v}^{2}$, ${\hat{\sigma }}_{\eta }^{2},$ and ${\hat{\bar{b}}}_{31}$ were set to 0, while the initial value of ${\hat{u}}_{\text{ref}}$ was set to 10 (when ${\hat{u}}_{\text{ref}}$ was a free variable). The true value ${\hat{G}}_{\mathrm{aa}}=0.025$ was used, such that estimates of $G_{\mathrm{bb}}$, $G_{\mathrm{ab}}$, $\sigma_{v}^{2},$ and $\sigma_{\eta }^{2}$ are found relative to $G_{\mathrm{aa}}=0.025$. Table 1 presents results for three cases, first for ${\hat{u}}_{\text{ref}}=u_{\text{ref}}=10$ (Case 1), second for ${\hat{u}}_{\text{ref}}$ as free variable (Case 2), and third for ${\hat{u}}_{\text{ref}}=11$ (Case 3), which is the expected initial value in the time series used. The estimates of $\sigma_{v}^{2}$ and $\sigma_{\eta }^{2}$ have in all cases rather large standard errors, and in some cases also large bias errors. What is more interesting is that the prediction errors $\Delta_{30}^{\text{error}}{\hat{\bar{a}}}_{t}\%$ and $\Delta_{30}^{\text{error}}{\hat{\bar{b}}}_{t}\%$ are small in Case 1 (with the correct value ${\hat{u}}_{\text{ref}}=10)$. In Case 2 (with ${\hat{u}}_{\text{ref}}$ as a free variable), $\Delta_{30}^{\text{error}}{\hat{\bar{a}}}_{t}\%$ has a large standard error. With ${\hat{u}}_{\text{ref}}=11$ (Case 3), $\Delta_{30}^{\text{error}}{\hat{\bar{a}}}_{t}\%$ has a large bias error. In this case also the estimates of $G_{\mathrm{bb}}$, $G_{ab,}$ and $\sigma_{\eta }^{2}$ are clearly biased. In Case 2 and Case 3, the $\Delta_{30}^{\text{error}}{\hat{\bar{b}}}_{t}\%$ results are close to the results with the correct value ${\hat{u}}_{\text{ref}}=10$ (Case 1), as explained in Subsection 2.5.

Table 1 includes theoretical prediction error results based on Equation (7), ${\hat{\bar{a}}}_{t,\text{corr}}={\hat{\bar{a}}}_{t}‐{\hat{\bar{b}}}_{t}\left({\hat{u}}_{\text{ref}}‐u_{\text{ref}}\right)$. These are in all cases close to the results with ${\hat{u}}_{\text{ref}}=u_{\text{ref}}=10$ (Case 1).

The results for Case 1 and Case 2 are very much improved with population size N=10,000, while the standard errors in Case 3 were only marginally improved by an increased population size (Appendix S3). The results for ${\hat{u}}_{\text{ref}}$ in Case 2 were, for example, improved from 9.92±0.66 to 9.95±0.19.

As shown in Table 1, a large error in the assumed reference environment ${\hat{u}}_{\text{ref}}$ results in large errors in predicted changes in ${\bar{a}}_{t}$ over 30 generations (Case 3). Table 2 shows prediction results for more moderate errors in ${\hat{u}}_{\text{ref}}$, as well as for ${\hat{u}}_{\text{ref}}=11.75$ (expected mean value in optimization data), and they are all in accordance with Equation (7).

**TABLE 2:** Errors in predicted total relative change in ${\bar{a}}_{t}$ and ${\bar{b}}_{t}$ over 30 generations, as functions of the reference environment ${\hat{u}}_{\text{ref}}$ used in the optimization procedure

${\hat{u}}_{\mathrm{ref}}$	$\Delta_{30}^{\mathrm{error}}{\hat{\bar{a}}}_{t}\%$	$\Delta_{30}^{\mathrm{error}}{\hat{\bar{b}}}_{t}\%$
9.75	−16 ± 4	0 ± 3
10	1 ± 4	−1 ± 3
10.25	17 ± 4	0 ± 3
10.5	34 ± 6	−2 ± 3
11	68 ± 8	−4 ± 4
11.75	115 ± 14	−8 ± 6

**FIGURE 5:**
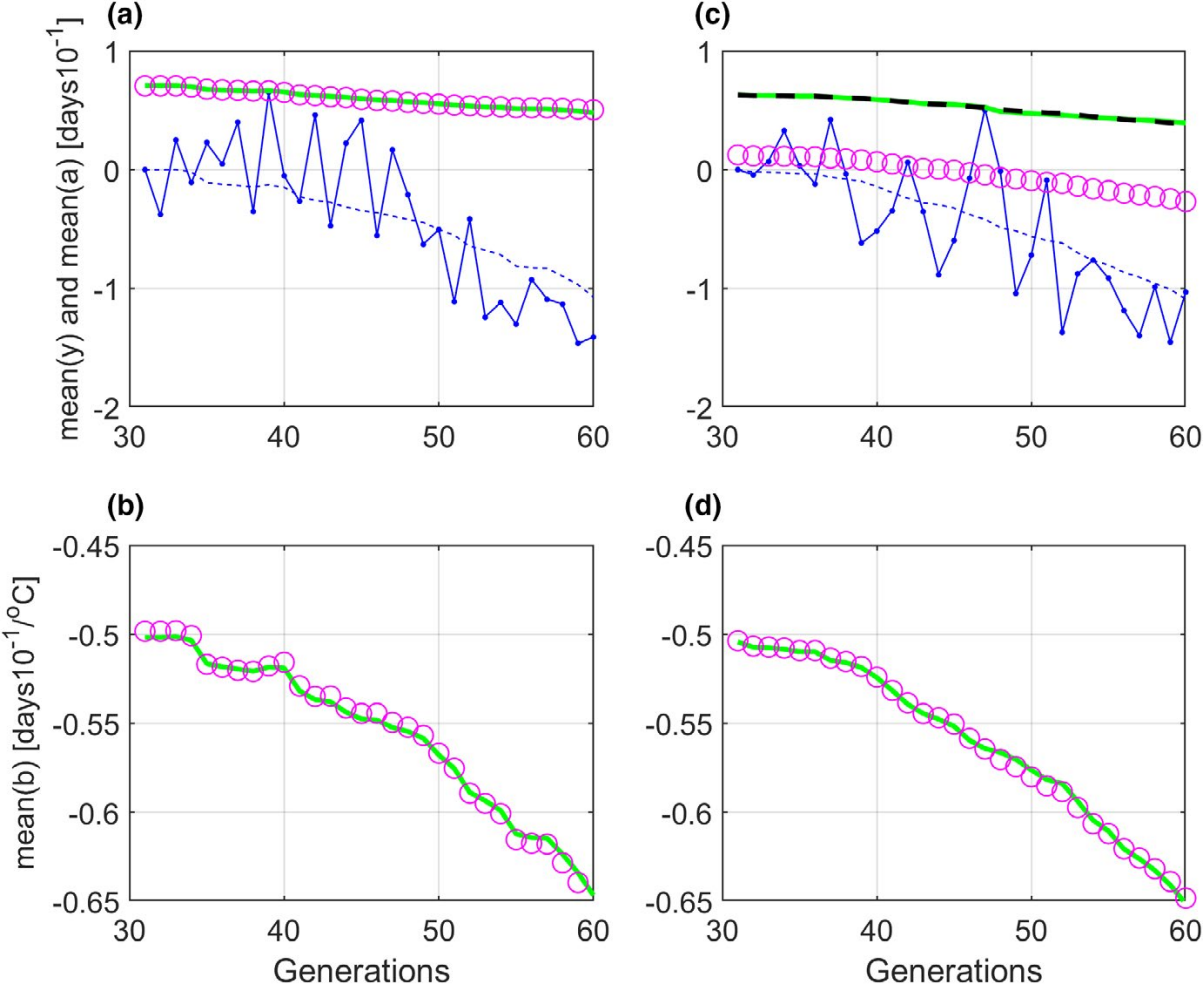
Typical responses for Case 1 and Case 3 in Table 1. True ${\bar{y}}_{t}$ values are shown by solid blue lines. All parameter values except ${\hat{u}}_{\text{ref}}$ and ${\hat{G}}_{\mathrm{aa}}=0.025$ were initially set to 0, which gave predictions ${\hat{\bar{y}}}_{t,\text{start}}=\text{cov}\left(W_{i,t},y_{i,t}\right)/{\bar{W}}_{t}$ as shown by dashed blue lines. Final predictions ${\hat{\bar{y}}}_{t}$ are shown by blue dots. True ${\bar{a}}_{t}$ and ${\bar{b}}_{t}$ responses are shown by green lines, while predictions ${\hat{\bar{a}}}_{t}$ and ${\hat{\bar{b}}}_{t}$ are shown by magenta circles. Panels a and b show results for Case 1 with ${\hat{u}}_{\mathrm{ref}}=10$ (true value), while panels c and d show results for Case 3 with ${\hat{u}}_{\mathrm{ref}}=11$. Here, the theoretical predictions ${\hat{\bar{a}}}_{t,\text{corr}}={\hat{\bar{a}}}_{t}‐{\hat{\bar{b}}}_{t}\left({\hat{u}}_{\text{ref}}‐u_{\text{ref}}\right)$ are included as black dashed line. Note that ${\bar{y}}_{31}={\hat{\bar{y}}}_{31}$ is set to 0, such that ${\bar{a}}_{31}=‐{\bar{b}}_{31}\left(u_{31}‐10\right)$ and ${\hat{\bar{a}}}_{31}=‐{\hat{\bar{b}}}_{31}\left(u_{31}‐{\hat{u}}_{\text{ref}}\right)$, where $u_{31}$ is not quite the same from realization to realization

Figure 5 shows predicted mean values ${\hat{\bar{y}}}_{t}$, ${\hat{\bar{a}}}_{t},$ and ${\hat{\bar{b}}}_{t}$, as compared to true mean values ${\bar{y}}_{t}$, ${\bar{a}}_{t},$ and ${\bar{b}}_{t}$, for Case 1 and Case 3 in Table 1. Note that ${\bar{y}}_{31}={\hat{\bar{y}}}_{31}$ is set to 0, and the zero point is thus not the same as in Figure 4, Panel a.

## 4 DISCUSSION

It is well documented that populations adjust to climate change by means of individual plasticity, but few reports on adaptation by means of genetically based microevolution caused by phenotypic selection (Merilä & Hendry, 2014). The main point in this article is that disentanglement of these separate effects requires that the reference environment $u_{\text{ref}}$ is defined, and that this should not be done arbitrarily. Instead, it should be based on the environment $u_{0}$ where the phenotypic variance is at a minimum (Definition 1 and Figure 1). This definition can be extended to multivariate cases.

As shown in a toy example, large errors in the estimated or assumed value ${\hat{u}}_{\text{ref}}$ may lead to large errors in predicted microevolutionary changes over time (Table 1 and Figure 5). Such large errors in ${\hat{u}}_{\text{ref}}$ may occur when the range of environmental data used for predictions is far from the mean value of the stochastic environment the population is adapted to.

In the toy example, the mean plasticity slope ${\bar{b}}_{t}$ is predicted quite well also when there is a large error in ${\hat{u}}_{\text{ref}}$, and one reason for this is that an error in ${\hat{G}}_{\mathrm{ab}}$ to some extent compensates for the error in ${\hat{u}}_{\text{ref}}$ (Table 1, Case 3). This does not, however, prevent a large error in the predicted mean intercept ${\hat{\bar{a}}}_{t}$ (Equation (7)).

Although the plastic response to climate change is a result of individual plasticity, it should be noted that individual traits do not determine the environmental value $u_{0}$ in Definition 1, but instead the phenotypic variance (Figure 1). Similarly, individual traits do not enter into the prediction Equations (6a, 6b), but instead the individual phenotypic and fitness values (Figure 4).

In theoretical studies, it is often assumed that the environmental variable is scaled such that $u_{\text{ref}}=u_{0}=0$ (Chevin & Lande, 2015; Lande, 2009). This can be done also in databased applications, provided that $u_{0}$ is known, and that the correction term in Definition 1 is 0.

The toy example used in the simulations is a simplification of reality because changing spring temperatures affect fitness in a complex way (Bowers et al., 2016). It still suffices to show that the reference environment together with initial mean trait and parameter values can be estimated from environmental, phenotypic, and fitness data, by use of the prediction error minimization method in Figure 2. The simulations make use of an environmental trend, as in noisy temperature trends caused by climate change (Figure 3), and a correct reference environment then results in quite good predictions of changes in mean traits over time (Table 1, Case 1). Although these predictions are based on parameter estimation, all the separate parameter estimates as such are not especially good, although they were considerably improved when the population size was increased from 100 to 10,000 (Appendix S3). The reference environment can also be estimated, but with large standard errors, especially for small population sizes, and this results in a correspondingly large standard error in predicted change in mean intercept ${\bar{a}}_{t}$ over time (Table 1, Case 2). An estimated reference environment may still be a better choice than use of an initial environmental value in a recorded time series, or the mean value, which may give large errors in predicted changes in mean traits (Table 1, Case 3). Another alternative may be to use the mean value of a past stationary stochastic environment, which the population is judged to have been fully adapted to.

It is here assumed that the genetic relationship matrix is an identity matrix, and the simulation results are obtained by use of a very simple system and a prediction error minimization method. However, the fact that errors in the reference environment may cause large errors in predictions of microevolution, as discussed in Subsection 2.5, is a generic problem. Independent of prediction method and the complexity of the model, an error in the reference environment implies that an erroneous model is fitted to the input–output data, and that must inevitably result in prediction errors. An alternative view, when ${\hat{u}}_{\text{ref}}\neq u_{\text{ref}}$ is constant (Case 3), is that the tuning model in Figure 2 still uses the correct value of $u_{\text{ref}}$, but then with an error term $u_{\text{ref}}‐{\hat{u}}_{\text{ref}}$ added to the environmental input. In order to minimize $\sum_{t=1}^{T}{\left({\bar{y}}_{t}‐{\hat{\bar{y}}}_{t}\right)}^{2}$, this input error must as good as possible be compensated by errors in estimated parameter values, resulting in prediction errors. Note that this argument is independent of the specific parametrizations used in the microevolutionary system and tuning model in Figure 2. It is in any case no reason to believe that prediction errors caused by ${\hat{u}}_{\text{ref}}\neq u_{\text{ref}}$ will disappear in cases where the genetic relationship matrix is not a unity matrix, and when other parameter estimation and mean trait prediction methods are used. A more specific argument regarding BLUP/REML parameter estimation is given in Appendix S4. It must thus be expected that predictions of microevolutionary changes over time depend on the chosen reference environment, and such predictions cannot therefore be trusted unless the chosen reference environment can be trusted. Exceptions are here cases with a constant mean plasticity slope, where the change in mean reaction norm intercept per generation according to Equation (5) is independent of the reference environment. This implies that a nearly constant mean plasticity slope must be expected to result in small errors in the predicted changes in the mean intercept, also if there is an error in the reference environment.

## CONFLICT OF INTEREST

The author declares no conflict of interest.

## AUTHOR CONTRIBUTION

**Rolf Ergon:** Conceptualization (equal); Data curation (equal); Formal analysis (equal); Funding acquisition (equal); Investigation (equal); Methodology (equal); Project administration (equal); Resources (equal); Software (equal); Supervision (equal); Validation (equal); Visualization (equal); Writing – original draft (equal).

## Supporting information

Supplementary Material

## Data Availability

MATLAB code for simulations is given in Supplementary Material archived in *bioRxiv*, https://doi.org/10.1101/2022.01.07.475361.
