# Supplementary material for: The important choice of reference environment in microevolutionary climate response predictions
Source: Ecol Evol. 2022 Apr 17;12(4):e8836. doi: 10.1002/ece3.8836 (PMC9013856; doi:10.1002/ece3.8836)
Supplement: Supplementary file 1 — Supplementary Material [file ECE3-12-e8836-s001.docx]

# Appendix S1. Derivation of prediction equations

The goal is to find Equations (6a,b) for incremental changes ${\Delta\bar{a}}_{t}=\bar{a}_{t+1}-\bar{a}_{t}$ and

$\Delta\bar{b}_{t}=\bar{b}_{t+1}-\bar{b}_{t}$, and thus also ${\Delta\bar{y}}_{t}=\bar{y}_{t+1}-\bar{y}_{t}$, by means of a common selection gradient $\beta_{y,t}$. Note that these equations implicitly assume that the additive genetic relationship matrix $\boldsymbol{A}_{t}$ in Fig. 2 is a unity matrix, if not the genetic relationships will simply be ignored.

As stated in the main text, a fundamental equation for mean trait predictions follows from Equation (2a) as

| $\Delta\bar{y}_{t}={\Delta\bar{a}}_{t}+\Delta\bar{b}_{t}\left( u_{t+1}-u_{ref} \right)+\bar{b}_{t}\Delta u_{t},$ | (5) |
| --- | --- |

where $\Delta u_{t}=u_{t+1}-u_{t}$. From this follows that the value of $u_{ref}$ has nothing to say in special cases with constant mean plasticity slopes, i.e., when $\Delta\bar{b}_{t}=0$. In such cases we simply have $\Delta\bar{y}_{t}={\Delta\bar{a}}_{t}+\bar{b}\Delta u_{t}$, where $\bar{b}$ is constant, or only $\Delta\bar{y}_{t}=\bar{b}\Delta u_{t}$ if $\bar{a}_{t}$ does not evolve.

In the general case, ${\Delta\bar{a}}_{t}$ and $\Delta\bar{b}_{t}$ are found from Equations (6a,b), as derived below. Assuming a constant environment, i.e., for $\Delta u_{t}=0$, Equation (5) gives

$\Delta\bar{y}_{t}^{c}={\Delta\bar{a}}_{t}+\Delta\bar{b}_{t}\left( u_{t+1}-u_{ref} \right)$, which by means of the breeder’s equation (Lande, 1979) also can be written as

| $\Delta\bar{y}_{t}^{c}=G_{yy,t}^{c}\beta_{y,t}=G_{yy,t}^{c}\frac{1}{\bar{W}_{t}} P_{yy,t}^{-1}cov\left( W_{i,t},y_{i,t} \right),$ | (A1) |
| --- | --- |

where $\beta_{y,t}$ is the selection gradient, while $G_{yy,t}^{c}$and $P_{yy,t}$ are the additive genetic and phenotypic variances. Here, $W_{i,t}$ is the individual fitness, with population mean value $\bar{W}_{t}$, while $y_{i,t}$ is the individual phenotypic value. We must assume that values of $u_{t+1}-u_{ref}$, $y_{i,t}$ and $W_{i,t}$ are available.

In order to find expressions for ${\Delta\bar{y}}_{t}$, ${\Delta\bar{a}}_{t}$ and $\Delta\bar{b}_{t}$, we may express ${\Delta\bar{a}}_{t}$ and $\Delta\bar{b}_{t}$ by means of $\beta_{y,t}$ according to

| $\left[ \begin{matrix} {\Delta\bar{a}}_{t} \\ {\Delta\bar{b}}_{t} \\ \Delta\bar{y}_{t}^{c} \end{matrix} \right]=\boldsymbol{G}_{aby,t}\left[ \begin{matrix} 0 \\ 0 \\ \beta_{y,t} \end{matrix} \right],$ | (A2) |
| --- | --- |

where the $\boldsymbol{G}_{aby,t}$ matrix is found via the linear transformation of the vector $\left[ \begin{matrix} a_{i,t} & b_{i,t} \end{matrix} \right]^{T}$ onto the vector $\left[ \begin{matrix} a_{i,t} & b_{i,t} & y_{i,t} \end{matrix} \right]^{T}$. By use of Equation (1) we find (using $u_{t}^{'}=u_{t}-u_{ref}$)

| $\left[ \begin{matrix} a_{i,t} \\ b_{i,t} \\ y_{i,t} \end{matrix} \right]=\left[ \begin{matrix} 1 & 0 \\ 0 & 1 \\ 1 & u_{t}^{'} \end{matrix} \right]\left[ \begin{matrix} a_{i,t} \\ b_{i,t} \end{matrix} \right]+\left[ \begin{matrix} 0 \\ 0 \\ v_{i,t}+\eta_{i,t}u_{t}^{'} \end{matrix} \right],$ | (A3) |
| --- | --- |

such that the additive genetic covariance matrix of $\left[ \begin{matrix} a_{i,t} & b_{i,t} & y_{i,t} \end{matrix} \right]^{T}$ specific to the environment $u_{t}^{'}$ becomes

| $\boldsymbol{G}_{aby,t}\left( u_{t}^{'} \right)=\left[ \begin{matrix} 1 & 0 \\ 0 & 1 \\ 1 & u_{t}^{'} \end{matrix} \right]\left[ \begin{matrix} G_{aa} & G_{ab} \\ G_{ab} & G_{bb} \end{matrix} \right]\left[ \begin{matrix} 1 & 0 & 1 \\ 0 & 1 & u_{t}^{'} \end{matrix} \right]$  $=\left[ \begin{matrix} G_{aa} & G_{ab} & G_{aa}+G_{ab}u_{t}^{'} \\ G_{ab} & G_{bb} & G_{ab}+G_{bb}u_{t}^{'} \\ G_{aa}+G_{ab}u_{t}^{'} & G_{ab}+G_{bb}u_{t}^{'} & G_{aa}+G_{ab}u_{t}^{'}+G_{ab}u_{t}^{'}+G_{bb}u_{t}^{'2} \end{matrix} \right]$. | (A4) |
| --- | --- |

From Equations (A1), (A3) and (A4) follow

| ${\Delta\bar{a}}_{t}=\left( G_{aa}+G_{ab}u_{t}^{'} \right)\frac{1}{\bar{W}_{t}} P_{yy,t}^{-1}cov\left( W_{i,t},y_{i,t} \right),$ | (A5a) |
| --- | --- |

and

| ${\Delta\bar{b}}_{t}=\left( G_{ab}+G_{bb}u_{t}^{'} \right)\frac{1}{\bar{W}_{t}} P_{yy,t}^{-1}cov\left( W_{i,t},y_{i,t} \right),$ | (A5b) |  |
| --- | --- | --- |

where

| $P_{yy,t}=G_{aa}+\sigma_{v}^{2}+2G_{ab}u_{t}^{'}+\left( G_{bb}+\sigma_{\eta}^{2} \right)u_{t}^{'2}.$ | (A5c) |
| --- | --- |

Since only a single environmental value is involved in Equations (A5a,b), they are valid in any environment, also if it varies with time¸ and $\Delta\bar{y}_{t}$ thus follows by inserting $\Delta\bar{a}_{t}$ and $\Delta\bar{b}_{t}$ from Equations (A5a,b) into Equation (5). For computations of $\bar{a}_{t}$and $\bar{b}_{t}$, and thus $\bar{y}_{t}$, initial values of $\bar{a}_{t}$ and $\bar{b}_{t}$ are needed.

Alternatively, $\Delta\bar{y}_{t}$ is found by inserting Equations (A5a,b) into Equation (5),

| ${\Delta\bar{y}}_{t}=G_{yy,t}\frac{1}{\bar{W}_{t}}P_{yy,t}^{-1}cov\left( W_{i,t},y_{i,t} \right)+\bar{b}_{t}\Delta u_{t},$ | (A6a) |
| --- | --- |

where

| $G_{yy,t}=G_{aa}+G_{ab}u_{t}^{'}+G_{ab}u_{t+1}^{'}+G_{bb}u_{t}^{'}u_{t+1}^{'}.$ | (A6b) |
| --- | --- |

Note that $G_{yy,t}^{c}$ in Equation (A1) follows from $G_{yy,t}$according to Equation (A6b) by setting $u_{t+1}^{'}=u_{t}^{'}$, i.e., under the assumption of a constant environment. The same expression for $G_{yy,t}^{c}$ also follows from Equation (A4).

# Appendix S2. Results with modeling error

Simulations with a true three-trait reaction norm model according to Equation (8), i.e., with a perception trait in addition to the intercept and plasticity traits (Ergon & Ergon, 2017), but with a two-trait tuning model, gave increased prediction errors as compared to results in Table 1. The population size was $N=100$. Simulations with $G_{cc}=G_{aa}=0.025$ and the true value $u_{ref}=10$, gave the following results: With $G_{ac}=0$ the prediction errors were

$\Delta_{30}^{error}{\hat{\bar{a}}}_{t}\%=-14\pm8$ and $\Delta_{30}^{error}{\hat{\bar{b}}}_{t}\%=2\pm7$. With $G_{ac}=-0.01$ the results were $\Delta_{30}^{error}{\hat{\bar{a}}}_{t}\%=-39\pm7$ and $\Delta_{30}^{error}\hat{b}_{t}\%=-2\pm4$, while $G_{ac}=0.01$ gave $\Delta_{30}^{error}{\hat{\bar{a}}}_{t}\%=9\pm8$ and $\Delta_{30}^{error}{\hat{\bar{b}}}_{t}\%=0\pm5$. Note that $\Delta_{30}^{error}\hat{b}_{t}\%$ is very much the same for all values of $G_{ac}$.

Typical responses are shown in Fig. A1. Note especially that the change in $\bar{a}_{t}$ over time for $G_{ac}\leq0$ is underestimated, which indicates that modeling errors in general may result in underestimated changes in $\bar{a}_{t}$. This should be compared with the results for Case 3 in Table 1, where changes in $\bar{a}_{t}$ are overestimated.


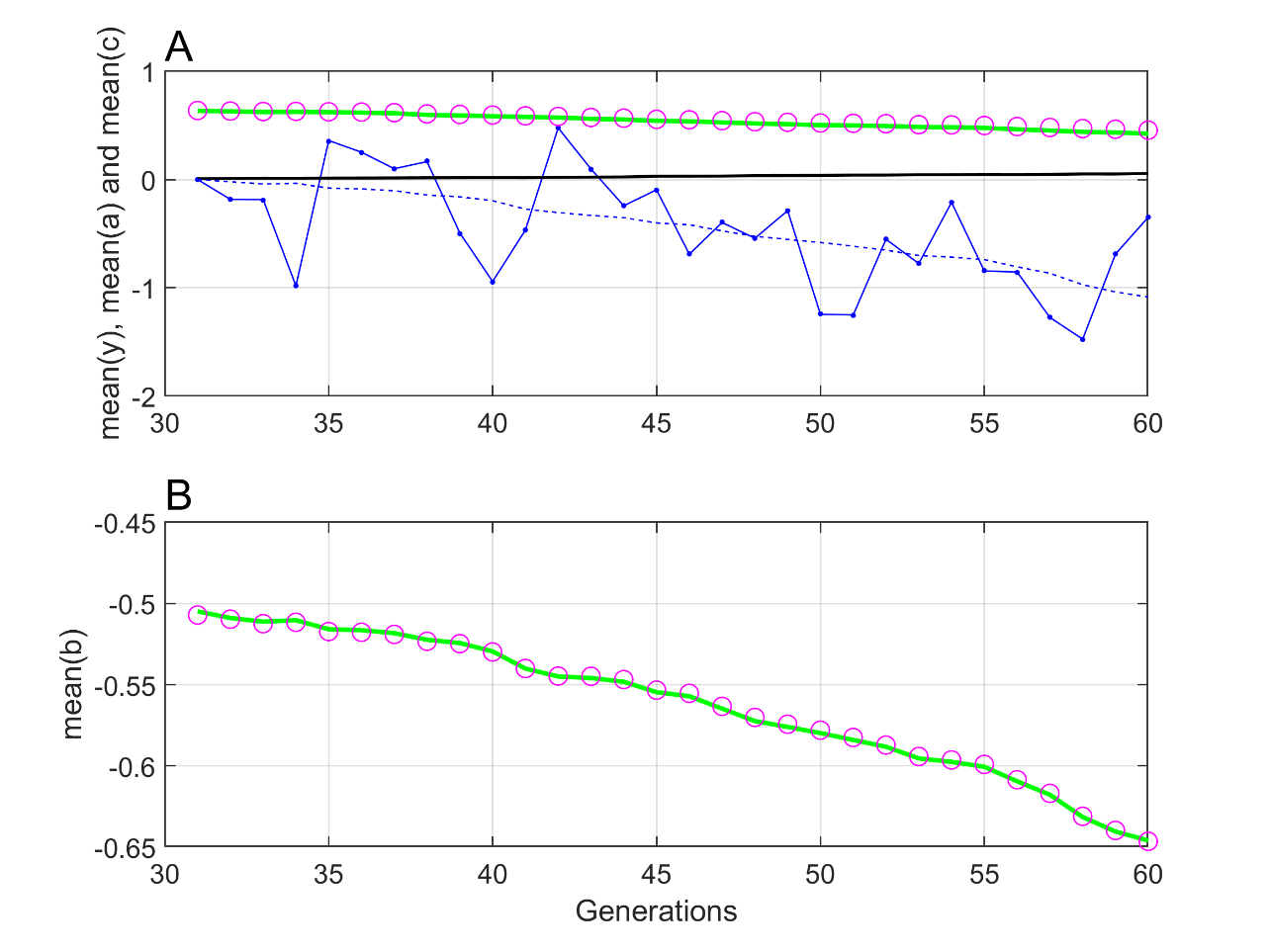


**Figure A1.** Typical responses as for Case 1 in Fig. 5, Panels A and B, but with a true three-trait model according to Equation (8) with $G_{cc}=G_{aa}=0.025$ and $G_{ac}=0$. The true response $\bar{c}_{t}-10$ is shown by black line.

# Appendix S3. Results with increased population size

Table A1 shows results as in Table 1, but with population size $N=10,000$. Note especially that $\sum\varepsilon_{t,final}^{2}$ in Case 2 is very much reduced when the population is increased, with the result that the standard error in $\hat{u}_{ref}$ is reduced.

**Table A1**. Estimation and prediction results as in Table 1, but with population size $N=10,000$. In Case 3, 16% of the simulations were discarded because

$\sum\varepsilon_{t,final}^{2}>0.001$.

| Parameter  etc. | True  value | Results  Case 1 | Results  Case 2 | Results  Case 3 |
| --- | --- | --- | --- | --- |
| $\hat{G}_{bb}$ | $0.01$ | $0.0100\pm0.0003$ | $0.0103\pm0.0023$ | $0.0064\pm0.0017$ |
| $\hat{G}_{ab}$ | $0$ | $0.0006\pm0.0033$ | $0.0002\pm0.0029$ | $0.0068\pm0.0015$ |
| $\hat{\sigma}_{v}^{2}$ | $0.025$ | $0.0269\pm0.0064$ | $0.0277\pm0.0146$ | $0.0256\pm0.0034$ |
| $\hat{\sigma}_{\eta}^{2}$ | $0.01$ | $0.0103\pm0.0021$ | $0.0102\pm0.0026$ | $0.0161\pm0.0027$ |
| ${\hat{\bar{b}}}_{31}$ | $-$ | $-0.4958\pm0.0068$ | $-0.4962\pm0.0086$ | $-0.4969\pm0.0089$ |
| $\hat{u}_{ref}$ | $10$ | 1$0$ | $9.9483\pm0.1921$ | $11$ |
| $\sum\varepsilon_{t,final}^{2}$ | $-$ | ${10}^{-5}(0\pm2$) | ${10}^{-5}(1\pm2)$ | ${10}^{-5}(10\pm10)$ |
| $\Delta_{30}^{error}{\hat{\bar{a}}}_{t}\%$ | $-$ | $0\pm1$ | $-3\pm12$ | $68\pm6$ |
| $\Delta_{30}^{error}{\hat{\bar{b}}}_{t}\%$ | $-$ | $0\pm1$ | $0\pm1$ | $-4\pm3$ |
| $\Delta_{30,corr}^{error}{\hat{\bar{a}}}_{t}\%$ | $-$ | $0\pm1$ | $0\pm1$ | $4\pm4$ |

# Appendix S4. BLUP/REML parameter estimation

The reaction norm model in Equation (1) can by use of Equation (2a) be written as

| $y_{i,t}=\bar{y}_{t}+a_{i,t}-\bar{a}_{t}+\left( b_{i,t}-\bar{b}_{t} \right)(u_{t}-u_{ref})+v_{i,t}+\eta_{i,t}(u_{t}-u_{ref}).$ | (A7a) |
| --- | --- |

From this follows the random mixed model for the population,

| $\boldsymbol{y}_{t}=\boldsymbol{1}_{N}\bar{y}_{t} +\left[ \begin{matrix} \boldsymbol{I}_{N} & \boldsymbol{U}_{t} \end{matrix} \right]\left[ \begin{matrix} \boldsymbol{a}_{t}^{'} \\ \boldsymbol{b}_{t}^{'} \end{matrix} \right]+\left[ \begin{matrix} \boldsymbol{I}_{N} & \boldsymbol{U}_{t} \end{matrix} \right]\left[ \begin{matrix} \boldsymbol{v}_{t} \\ \boldsymbol{\eta}_{t} \end{matrix} \right],$ | (A7b) |
| --- | --- |

where $\boldsymbol{a}_{t}^{'}=\boldsymbol{a}_{t}\boldsymbol{-}\boldsymbol{1}_{N}\bar{a}_{t}$ and $\boldsymbol{b}_{t}^{'}=\boldsymbol{b}_{t}\boldsymbol{-}\boldsymbol{1}_{N}\bar{b}_{t}$ (where $\boldsymbol{1}_{N}$ is an $N\times1$ vector of ones). Here, $\boldsymbol{y}_{t}$**,** $\boldsymbol{a}_{t}\boldsymbol{,}$ $\boldsymbol{b}_{t}$ $\boldsymbol{a}_{t}^{'}$, $\boldsymbol{b}_{t}^{'}$, $\boldsymbol{v}_{t}$ and $\boldsymbol{\eta}_{t}$ are $N\times1$ vectors of individual values at time $t$, while $\bar{y}_{t}$ is the scalar valued fixed effect. The environmental input matrix is $\boldsymbol{U}_{t}=(u_{t}-u_{ref})\boldsymbol{I}_{N}$, where $\boldsymbol{I}_{N}$ is the $N\times N$ unity matrix. The expected values $E\left[ \boldsymbol{a}_{t}^{'} \right]$, $E\left[ \boldsymbol{b}_{t}^{'} \right]$, $E\left[ \boldsymbol{v}_{t} \right]$ and $E\left[ \boldsymbol{\eta}_{t} \right]$ are all zero by definition (Ch. 26, Lynch and Walsh, 1998).

The starting point for REML parameter estimation based on the model (A7b) is the phenotypic population covariance matrix $\boldsymbol{V}_{t}\boldsymbol{=}E\left[ \left( \boldsymbol{y}_{t}-\boldsymbol{1}_{N}\bar{y}_{t} \right)\left( \boldsymbol{y}_{t}-\boldsymbol{1}_{N}\bar{y}_{t} \right)^{T} \right]$ (Ch. 27, Lynch and Walsh, 1998). For the model in Equation (1) this covariance matrix is

| $\boldsymbol{V}_{t}=E\left[ \left( \boldsymbol{a}_{t}-\boldsymbol{1}_{N}\bar{a}_{t}+\boldsymbol{v}_{t}+\left( \boldsymbol{b}_{t}-{\boldsymbol{1}_{N}\bar{b}}_{t} \right)\left( u_{t}-u_{ref} \right)\boldsymbol{+}\boldsymbol{w}_{t}\left( u_{t}-u_{ref} \right) \right)\left( \cdot\right)^{T} \right]$  $=\left( G_{aa}\boldsymbol{+}{2G}_{ab}\left( u_{t}-u_{ref} \right)\boldsymbol{+}G_{bb}{(u_{t}-u_{ref})}^{2} \right)\boldsymbol{A}_{t}\boldsymbol{+}\left( \sigma_{v}^{2}+{(u_{t}-u_{ref})}^{2}\sigma_{\eta}^{2} \right)\boldsymbol{I}_{N}$, | (A8) |
| --- | --- |

where $\boldsymbol{A}_{t}$ is the genetic relationship matrix (Ch. 27, Lynch and Walsh, 1998). From Equation (A8) follows that REML parameter estimation results depend on $u_{ref}$, such that errors in the reference environment give errors in estimated parameter values. As in parameter estimation based on Equations (6a,b), we can in REML estimation set $G_{aa}$ to any value, and estimate other parameters in relation to that. It follows from Equation (A8) that we cannot find $\hat{G}_{ab}$ separate from $\hat{G}_{bb}$, and $\hat{\sigma}_{v}^{2}$ separate from $\hat{\sigma}_{\eta}^{2}$, unless data for several different values of $u_{t}$ are used. This may be compared with the PEM method in Fig. 2, as used in the simulations, where data from all available generations are utilized.
